# Strategies supporting sustainable prescribing safety improvement interventions in English primary care: a qualitative study

**DOI:** 10.3399/BJGPO.2021.0109

**Published:** 2021-08-18

**Authors:** Azwa Shamsuddin, Mark Jeffries, Aziz Sheikh, Libby Laing, Nde-Eshimuni Salema, Anthony J Avery, Antony Chuter, Justin Waring, Richard N Keers

**Affiliations:** 1 Centre for Medical Informatics, Usher Institute, University of Edinburgh, Edinburgh, UK; 2 Centre for Pharmacoepidemiology and Drug Safety, Division of Pharmacy and Optometry, School of Health Sciences, University of Manchester, Manchester, UK; 3 National Institute for Health Research Greater Manchester Primary Care Patient Safety Translational Research Centre, University of Manchester, Manchester Academic Health Sciences Centre, Manchester, UK; 4 Division of Primary Care, School of Medicine, University of Nottingham, Nottingham, UK; 5 School of Social Policy, Health Services Management Centre, University of Birmingham, Birmingham, UK; 6 Pharmacy Department, Greater Manchester Mental Health NHS Foundation Trust, Manchester, UK

**Keywords:** general practice, implementation, patient safety, prescribing safety, primary health care, sustainability

## Abstract

**Background:**

While the use of prescribing safety indicators (PSI) can reduce potentially hazardous prescribing, there is a need to identify actionable strategies for the successful implementation and sustainable delivery of PSI-based interventions in general practice.

**Aim:**

To identify strategies for the successful implementation and sustainable use of PSI-based interventions in routine primary care.

**Design & setting:**

Qualitative study in primary care settings across England.

**Method:**

Anchoring on a complex pharmacist-led IT-based intervention (PINCER) and clinical decision support (CDS) for prescribing and medicines management, a qualitative study was conducted using sequential, multiple methods. The methods comprised documentary analysis, semi-structured interviews, and online workshops to identify challenges and possible solutions to the longer-term sustainability of PINCER and CDS. Thematic analysis was used for the documentary analysis and stakeholder workshops, while template analysis was used for the semi-structured interviews. Findings across the three methods were synthesised using the RE-AIM (reach, efficacy, adoption, implementation, and maintenance) framework.

**Results:**

Forty-eight documents were analysed, and 27 interviews and two workshops involving 20 participants were undertaken. Five main issues were identified, which aligned with the adoption and maintenance dimensions of RE-AIM: fitting into current context (adoption); engaging hearts and minds (maintenance); building resilience (maintenance); achieving engagement with secondary care (maintenance); and emphasising complementarity (maintenance).

**Conclusion:**

Extending ownership of prescribing safety beyond primary care-based pharmacists, and achieving greater alignment between general practice and hospital prescribing safety initiatives, is fundamental to achieve sustained impact of PSI-based interventions in primary care.

## How this fits in

The use of PSI has been shown to be effective at reducing potentially hazardous prescribing in general practice. There, however, remains a need to identify actionable strategies to maximise the reach and achieve longer-term sustainability of PSI-based interventions. Previous studies have shown the following to be important: availability of resources; simplifying the use of interventions; avoiding alert fatigue; pharmacist embedment; and improving the specificity of PSIs. This qualitative study synthesises findings from a documentary analysis, interviews, and workshops with local, regional, and national primary care stakeholders, to draw lessons from the implementation of a complex PINCER and CDS. This results in five strategies centering around engagement with the primary care team and alignment of policies to achieve sustainable use of PSI-based interventions.

## Introduction

**Figure 1.**
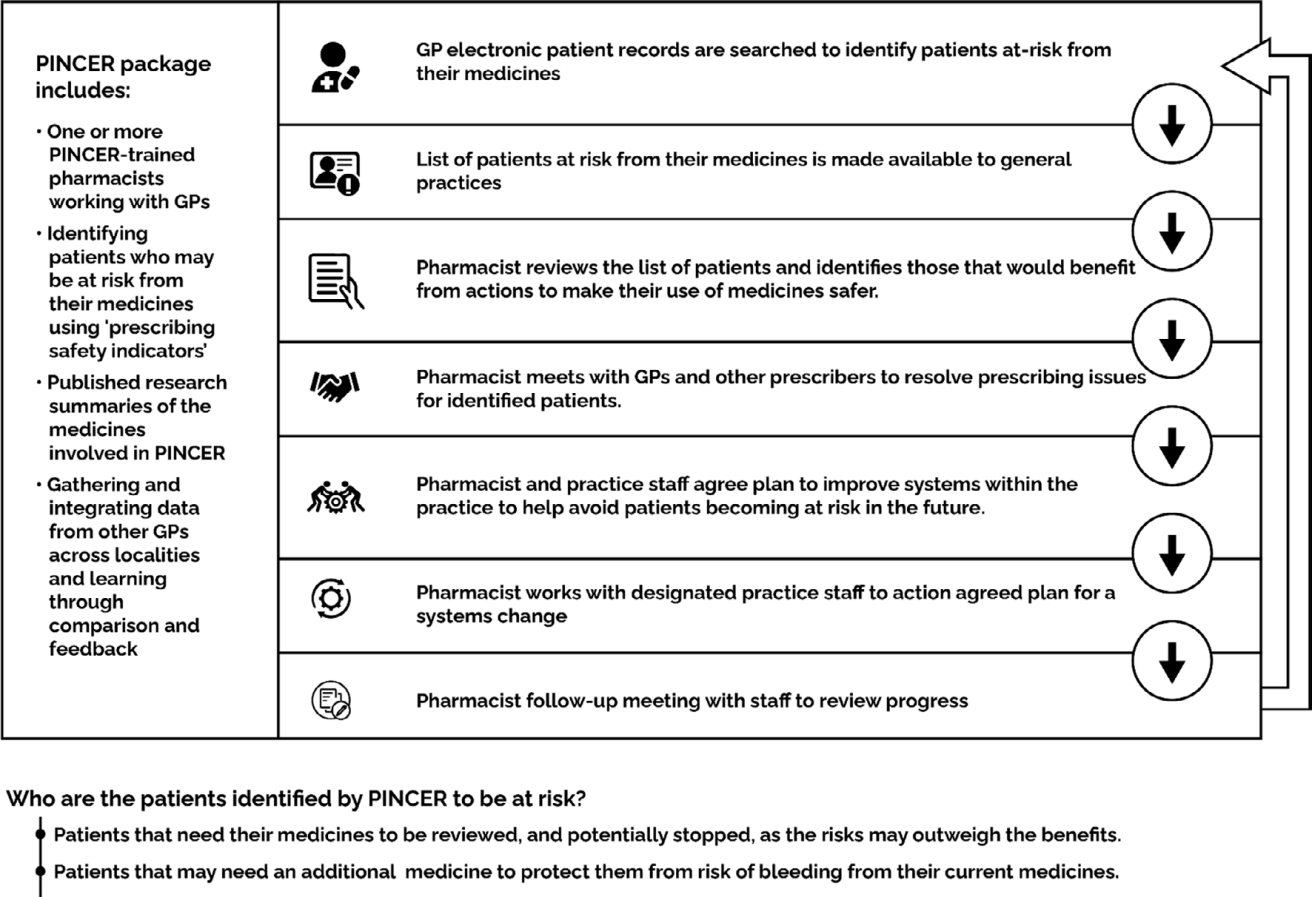
Summary of pharmacist-led IT-based intervention (PINCER) in general practice

**Figure 2.**
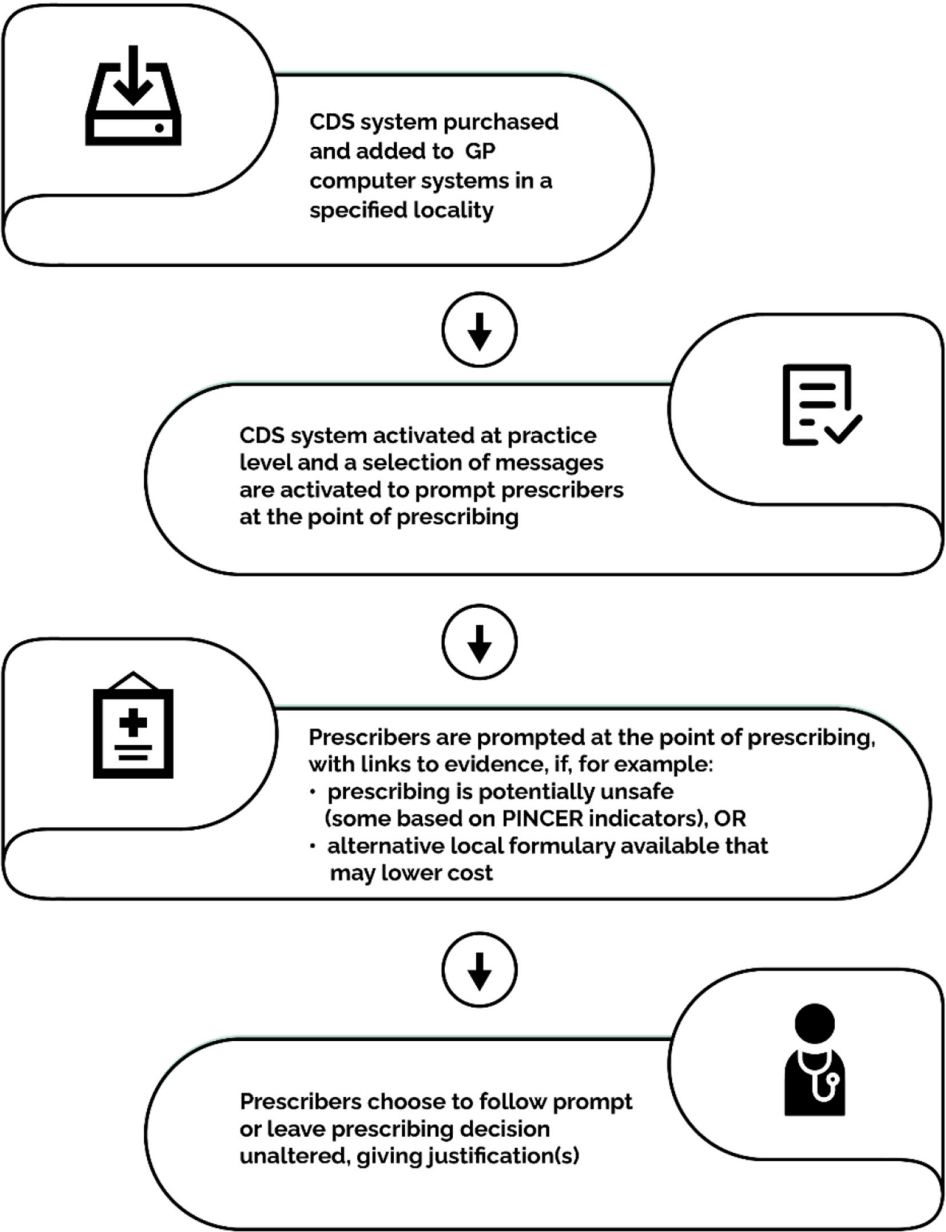
Summary of clincial decision support (CDS) interventions in general practice

Prescribing and medication errors are common in primary care. Although many are not serious, they overall — as a function of the substantial volume of prescribing in this setting — are responsible for considerable potentially avoidable harm.^1^ There is evidence from the authors' work and others that focusing on PSI offers an opportunity to reduce the frequency of these errors, which may also translate into improved health outcomes.^2,3^ These indicators have been embedded in technology-enabled interventions to improve prescribing safety in primary care such as CDS and PINCER,^4^ both of which have demonstrated positive impact in reducing the rate of potentially hazardous prescribing in primary care.^2,4,5^ CDS systems are often used in primary care for support in decision making for prescribers at the point of prescribing. They provide patient-related information and clinical knowledge to enhance patient care, and may reduce medication error or the potential prescribing of inappropriate medications. Alternatively, PINCER is a pharmacist-led intervention that involves a search of patient records to identify those at risk of potentially hazardous prescribing and/or monitoring errors based on PSIs built into the software, and an educational outreach programme for general practice staff. Further details on how the PINCER and CDS interventions occur in general practice are outlined in Figures 1 and 2.

There remains, however, a need to better understand how these approaches, centred on CDS and expert pharmacist input, might be both scaled up and systematised into routine clinical practice. The avoiding patient harm through the application of prescribing safety indicators in English general practices (PRoTeCT) research programme aims to evaluate PINCER and CDS systems to respond to the international call made for evidence through the World Health Organization’s (WHO's) third global patient safety challenge: *‘* *Medication without harm* *’* ^6^ and national drivers.^7^ In this article, strategies are outlined for the successful sustainment, reach, and optimisation of PSI-based interventions in primary care by drawing lessons learnt from the longer-term implementation of PINCER and CDS across England.

## Method

A qualitative study was undertaken using sequential, multiple methods, comprising a documentary analysis, in-depth qualitative interviews, and participatory workshops. These three interlinked phases produced findings that were synthesised using the RE-AIM framework^8^ to consolidate learning on the various key factors that influence the sustained implementation of the PINCER and CDS interventions.

### Phase 1: Documentary analysis

A scoping search was conducted with the aim of identifying important factors influencing the sustainable adoption of PSI-based interventions in primary care. Ovid MEDLINE and Google Scholar were searched using the terms: 'healthcare information technolog*'; or 'primary care'; or 'prescribing safety'; or 'prescribing safety indicator*'; or 'implementation'; or 'evaluation', with no date restrictions up to September 2020. The eligibility criteria included documents that discussed implementation and/or sustainability of PSI-based interventions in primary care. To provide context, articles on the implementation or evaluation of similar interventions in primary care that aimed to improve prescribing safety, such as e-prescribing, medication reconciliation technology, and other interventions, were reviewed. Articles not in English were excluded.

Thirty-eight papers met the eligibility criteria and an additional 10 documents relating to publications from relevant professional bodies and reports on previous rollouts of the interventions (for example, from Academic Health Science Networks, clinical commissioning groups, and software developers) were suggested by topic experts within the research team and included to give a total of 48 documents. These consisted of peer reviewed research articles, reports to funders, NHS guidance and briefing documents, professional standards guidance, expert-written articles, and both analysed and unanalysed data from the PRoTeCT research team.

One author, a post-doctoral research fellow experienced in qualitative research, analysed the data thematically by extracting data on issues affecting implementation, sustainability, and reach of the interventions to produce thematic findings. This thematic analysis was completed in parallel with a review of the implementation science and adoption of complex interventions literature to ensure important themes in the data were considered alongside relevant theoretical frameworks. Thematic findings from the documentary analysis were used to generate the subsequent interview guide and to build on the initial coding framework for stakeholder interviews.

### Phase 2: Stakeholder interviews

Twenty-seven virtual semi-structured interviews were held via Microsoft Teams (in compliance with restrictions to travel and in-person meetings in the context of the COVID-19 pandemic). Interviews were conducted by a postdoctoral researcher with a nursing background outside of the UK. Participants were eligible if they had either had direct contact with, understood, and/or were in a position to influence wider or sustained adoption of the PINCER and CDS interventions at local, regional, or national levels.

Sampling began purposively using existing networks of the research team. It then proceeded to snowball sampling, building on the networks of participants and fulfilling the needs of the data in gaining the perspectives of significant stakeholders in prescribing safety in primary care until data saturation was reached at 27 interviews. Interviews were guided by a topic guide from a synthesis of the above-mentioned documentary analysis findings. Interviews lasted 20–90 min, were digitally audiorecorded, and transcribed by an approved transcribing service. Each participant was interviewed only once.

Analysis of interviews was data driven using template analysis^9^ and emerging key themes were mapped to the RE-AIM framework.^8^ NVivo (version 12) was used to manage the data and one author coded the data. The coding framework and nine transcripts were reviewed by a further author for second coding and internal validity. Key themes emerging from the data were discussed and agreed by the research team at regular meetings. Interview findings were presented at the stakeholder workshops for discussion and comments to generate strategies for scaling-up and enhancing sustainability.

### Phase 3: Stakeholder workshops

The aim of the workshops was to generate recommendations for optimising the use and impact of the CDS and PINCER interventions by discussing issues that affect the reach, adoption, and sustainable delivery of the interventions across primary care in England. Two stakeholder workshops were held 2 weeks apart, consisting of 11 participants in the first workshop and nine in the second workshop. Participants from the previous interview phase were invited via email and asked to disseminate the invitation email to their networks. Patient and public involvement (PPI) groups were also approached for participation and invitation emails were also sent to 952 PINCER users who had given consent to be contacted for research purposes. Participants in each workshop were assigned to groups of three or four and discussed three topics with facilitation by three authors. Topics of discussion for the first workshop were developed from findings of the interviews. The topics for the second workshop were developed from findings of the first workshop to further explore issues raised by participants alongside the interview data. From their discussion of each topic, participants in groups were asked to select three important points to bring to the main group discussion for selection and ranking between all participants.

Researcher field notes and workshop artefacts (notes from group discussions and results of ranking exercise) were analysed thematically and compared with findings of the documentary analysis and stakeholder interviews. Both workshops were conducted online via Microsoft Teams and lasted 2 hours each. Microsoft PowerPoint was used for presentations, Padlet was used for group tasks and discussion, and lastly, Mentimeter was used for the ranking exercise.

## Results

The findings revealed five main themes describing strategies supporting sustained impact of PSI-based interventions, four of which mapped to the ‘maintenance’ dimension of RE-AIM, with one strategy mapping to the ‘adoption’ dimension. A summary of the RE-AIM framework is provided in Table 1. These strategies are outlined below, ranked according to priority by workshop participants, and examples of evidence for these strategies are presented in Supplementary Table S1.

**Table 1.** RE-AIM framework^8^

**Dimension**	**Definition**	**Contextual level**
Reach	Proportion of the target population that participated in the intervention	Individual
Efficacy	Success rate if implemented as intended	Individual
Adoption	Proportion of settings, practices, and plans that will adopt this intervention	Organisational
Implementation	Extent to which the intervention is implemented as intended in the real world	Organisational
Maintenance	Extent to which a programme is sustained over time	Individual and organisational

### Strategy for success #1: Fitting PSI-based interventions into the current context

The data indicated that fitting PSI-based interventions into the current context is key to achieving maximum adoption. Context was perceived to be at the micro, meso, and macro levels, in that the intervention had to be easy for the practice team to use (micro level), be integrated into current IT systems (meso level), and align to local and/or national agendas (macro level).

Facilitating ease of use for practice staff was a shared view across interview and workshop participants with regards to both access and use of the intervention. In the participants’ view, this meant making the intervention as easy to use as possible so as not to disrupt the workflow:

*‘Anything that slows the consultation down isn’t often welcomed.* *’* (Interview participant, GP, professional body representative)

The importance of ease of use is further captured in the documentary analysis where it was argued, *‘even if the cited CDS interventions are transferable, usability challenges could limit impact on health processes and/or outcomes* *‘*.^10^

Making the intervention easy to use was closely related to interoperability or integration of the interventions to existing IT systems within the practice. Participants across the interviews and workshops believed integration of the intervention with existing IT systems would facilitate its use. In embedding the interventions into the IT system, the impact of the interventions need to align with existing local and national agendas to increase their visibility to policymakers. This alignment to policies could take a number of forms; for example, incentivised efforts or guidelines to best practice that would place the intervention as important to implement.

Additionally, workshop participants highlighted the importance of supporting local health providers to achieve local targets using the interventions. The support of local healthcare organisations was seen as essential alongside national funding for the interventions, rather than in place of, to ensure that each locality was sufficiently engaged and motivated to implement the interventions over time.

### Strategy for success #2: Engaging hearts and minds

Data from interviews and workshops clearly indicated the need to align the impact of the interventions with the professional values of stakeholders in order to achieve longer-term sustainability of PSI-based interventions, which mapped to the ‘maintenance’ dimension of RE-AIM. This alignment involved engaging both emotional and cognitive aspects, by making explicit the benefits of the interventions to the values of implementers and users. Similarly, workshop participants who were clinicians suggested using the intervention for planning and prioritisation of their daily workload to generate caseloads according to patient risk and matching clinical capacity:

*‘Clinicians have to be convinced that it is worth their while having it there and the irritations that it causes. If we knew for sure that it is saving X million pounds and where is that investment going back in to? How is that being helpful? If it is improving the quality of prescribing well let’s see the evidence of that being provided to us but I never see that* *.* *’* (Interview participant, GP, professional body representative)

Workshop and interview participants pointed out the importance of leadership in achieving longer-term engagement with the interventions. They envisioned PSI-based intervention ‘champions’ to be those in leadership or otherwise influential positions at both local and national levels, and were seen as vital at every level to achieve sustained engagement with the interventions by advocating for them. In the documentary analysis, multilevel buy-in was seen as vital for sustaining PSI-based interventions for the longer term, particularly in facilitating a cultural change in practice.^11^

### Strategy for success #3: Building resilience

Across the workshops and interviews, participants outlined the importance of taking a team approach across health care in implementing and sustaining PINCER and CDS to ensure they became part of everyday practice. Participants underscored the importance of engaging across professions within general practice in implementing the principles of prescribing safety. They perceived this as an important sustainability measure to maintain PSI-based interventions over time, particularly in withstanding fast-paced changes. Participants generally welcomed pharmacist-led interventions for prescribing safety, yet importantly pointed out concerns in deskilling and change of staff that could affect the sustainability of the intervention(s):

*‘...* *a concern is when the pharmacist is away, it all defaults back to the GPs again and then we have almost got deskilled to some extent because they have got the expertise and we haven’t* *.* *’* (Interview participant, GP, professional body representative)

Participants also claimed that impact was enhanced and risk reduction was increased with the involvement of all staff members compared with a focus on the pharmacist to implement PINCER. Further, participants suggested that involvement of all staff members with PINCER facilitated the embedment of the pharmacist within the practice team, a concern identified in previous PINCER publications.^12^

Workshop participants suggested that GPs were key in advocating pharmacists to patients, highlighting their knowledge and expertise to build a foundation of trust. Participants in interviews felt that patients were less receptive of a medication review with allied health professionals, especially if they were not familiar with the healthcare professional or aware of their competencies. From the workshops, PPI participants emphasised longer consultation appointments for medication reviews to explain the risks and benefits of medications to facilitate self-management and empowering of patients in their medicines management.

Workshop and interview data highlighted the need for adequate resources in terms of funding and time for training in engaging across the practice team, with PINCER perceived as a core patient safety intervention involving the role of all practice staff members. Workshop and documentary analysis data^4,13^ indicated that the ‘ideal’ scenario would be for PINCER searches to be centrally located without the need for additional software, further facilitating use and engagement across the practice team.

### Strategy for success #4: Achieving engagement with secondary care to align prescribing safety guidelines

The data revealed the need to engage with secondary care for alignment of prescribing safety guidelines in order to sustain PINCER and CDS. Although alignment with secondary care was mentioned by interview participants, this topic was augmented in the workshops, leading to a focus on secondary care in the second workshop to explore this theme in depth.

Participants identified the need to align the IT capabilities and infrastructure between primary and secondary care. Participants claimed that there was a mismatch in IT systems between secondary and primary care to align prescribing safety guidelines between the two sectors:

*‘…* *for PINCER to be able to get the hospital data, it needs some sort of shared care record, infrastructure, which I guess isn’t really available within practice systems at the moment.* *‘* (Interview participant, Researcher, PRoTeCT research team)

This effort would need to involve stakeholders of both sectors, including patients and the public, underscoring the need to involve multiple organisations, as similarly noted in the documentary analysis.^4,11,14^ The importance of ‘buy-in’ from various organisations was a recurrent theme in the data. Participants highlighted the need for involvement of key influencer bodies in supporting the interventions and/or structured medication reviews. This was also asserted to be essential in achieving nationwide training for PINCER with the engagement of higher education institutes and educational bodies in incorporating training at undergraduate and postgraduate levels, additionally supporting the alignment of primary and secondary care.

Participants highlighted the need to improve communication between primary and secondary care in optimising medications as there was the potential for missed communication regarding medications owing to workload. They also suggested that discussions needed to occur across the NHS regarding prescribing safety involving patients and healthcare providers, with PINCER training and development of indicators as part of this system-wide discussion to align guidelines for secondary and primary care.

### Strategy for success #5: Emphasising complementary use of PSI-based interventions

Several participants highlighted the need to emphasise the potential for complementary use of PSI-based interventions in enhancing patient safety, in order to address any decision by some organisations to implement one PSI-based intervention in favour of the other. Participants across workshops and interviews saw PINCER and CDS systems as complementary in enhancing prescribing safety:

*‘We describe it as being PINCER is your annual MOT of your car and* [CDS system] *is your dashboard, OK so* [CDS] *is able to tell you in the moment where your issues are and what is happening but your PINCER work is what you’re dipping in and out of every kind of three to six months* *.* *’* (Interview participant, primary care data manager)

Emphasising complementarity was similarly seen as a strategy for adoption and subsequent sustainability in the documentary analysis.^9,15^ An important approach to supporting complementary use of PSI-based interventions, as suggested by participants, was the publication of prescribing safety data to demonstrate the benefits and impact of concurrent use of PINCER and CDS. Publishing such data was also suggested to share learning and best practice between practices and regions, additionally acting as a driver to implement the interventions and/or structured medication reviews. Publishing prescribing safety data according to localities was suggested to be made available in the public domain, and hence should be developed in collaboration with PPI partners. The data to be published was suggested by participants to focus primarily on PINCER indicators and where localities sit within them to further support sustainability of the PSI-based interventions.

## Discussion

### Summary

Five key strategies have been successfully developed supporting the effective implementation, impact, and sustainable use of PSI-based interventions in primary care. The sequential study design has ensured that these strategies are grounded in the experiences and perceptions of key stakeholders at different levels of the healthcare system. As attention focuses on improving medication safety following the WHO's third global patient safety challenge, the findings directly address this call for action. This has been achieved by focusing on high-risk prescribing (PSIs) and delivering a resource that may be used by policymakers and healthcare managers to enhance prescribing safety in primary care, which can additionally be aligned with secondary care. Unpacking the five key strategies illuminates the importance of alignment of services, taking a team approach across the healthcare system, and sharing information in sustaining the delivery and impact of PSI-based interventions designed to improve prescribing safety. The role of support from organisations from local to national levels was also clear, in terms of resources and advocating the interventions by key healthcare leaders.

### Strengths and limitations

The study adopted a sequential, in-depth exploration of the issues relating to the optimisation and sustainability of PINCER and CDS from the available literature and stakeholder experiences. It used different methods to draw lessons to inform strategies designed to support the sustainable impact of PSI-based interventions in the longer term. The qualitative nature of the study enabled rich perspectives into the issues raised by participants to be explored and triangulation of multiple sources of data (documents, interviews, and workshops) corroborated analytical findings.^16^ The sequential approach to data collection (that is, documentary analysis, semi-structured interviews, and then workshops), with findings from one phase informing the next phase, further connected the emerging data and added rigour to the approach. The participants included stakeholders who varied in age and experience in primary care across multiple organisations, ranging from NHS England to professional and educational bodies and medicines optimisation teams, to ensure the relevance of findings to both research and practice.

The study also has important limitations. While several patient and public groups were contacted and invited to participate, only groups with particular interest in medication safety responded with interest, thus the views of wider patient and public groups were not included. Similarly, clinician participants in the interviews and workshops were often directly involved with medication safety interventions and were likely to recognise and understand the burden of drug-related harm, and this may, therefore, not be representative of wider professional groups. Additionally, the literature search for the documentary analysis was not a systematic review, thus some relevant literature may have been missed, although the risk of this was minimised by examining the reference lists of included studies and obtaining recommendations from the wider research team.

### Comparison with existing literature

The importance of a team approach to the delivery of primary care services is well known,^10,17^ although increasing evidence pointing to the role of the pharmacist in enhancing prescribing safety within general practice continues to emerge.^18–21^ Historically, scholars have suggested the integration of the pharmacist in primary care, using examples of pockets of primary care services with dedicated pharmacy input. Pharmacists within the Department of Veterans Affairs (VA) in the US have been reported to play a vital role within the healthcare team in providing recommendations to prescribers, collaborating with healthcare teams in secondary care, providing preventive medicine services, managing the VA drug formulary, and establishing the file structure and clinical pathways for prescribing in healthcare information technologies.^20^ A study in the UK placed pharmacists in general practices with roles ranging from patient consultations to managing long-term conditions, medicines optimisation, and reconciliation of discharge summaries.^22^ Positive experiences and decreased workloads were among the outcomes of the study; however, pharmacists in that study echoed the present study's findings in underscoring the need for further training and outlining clear job roles to fully function in primary care.^22^ Contemporary studies have highlighted the importance of involving the wider general practice team in prescribing safety interventions,^23–25^ supporting the present findings on taking a team approach across primary care to sustain PSI-based interventions. While the integration of pharmacists in general practice continues to be a positive development,^26,27^ the findings indicate that other healthcare professionals may lead interventions such as PINCER effectively. This could additionally expedite the adoption of services in areas where pharmacists are not yet available to support general practice owing to recruitment issues for the pharmacy profession in general practice.^28,29^ Taking a team approach with the training and implementation of PSI-based interventions importantly builds sustainability by not relying on particular individuals for the sustainability of the intervention by spreading ownership across healthcare professions.^30^

The findings also point to the need for collaborations of key influencer bodies and patient and public groups at all levels to support embedding of PSI-based interventions in general practices across England. At a local level it is vital that the trajectory of conversations between stakeholders^31^ reflects local needs and fosters ownership. Other studies that have explored changing prescribing practice in primary care also recognise the need for flexibility and tailoring strategies according to local resources in engaging general practices.^32,33^

A previous study has found that prescribers were more likely to engage with prescribing change interventions if they perceived it to be an important patient safety issue rather than one that focused on cost reduction.^33^ This is consistent with the findings on engaging the professional values of prescribers and practice staff members, in this case on the quality of patient care. Further engagement and impact was seen in studies that used local leaders as champions of prescribing change interventions.^34–36^ A study investigating the role of champions in primary care change found that champions at both the project and organisational levels were critical in driving innovation-specific and transformative change,^35^ echoing participants in the present study in suggesting the need for champions at local and national levels. A commonality in these studies is the additional finding of the effectiveness of audit and feedback approaches in pushing forward changes in prescribing practice.

Peer-comparison data were found as a significant motivator for changing prescribing practices, even without any financial incentives, although this has been suggested to maximise impact.^32,37^ Collecting and showing data on the magnitude of the problem, seen here as potentially hazardous prescribing, is an important part of convincing stakeholders of the urgency of the problem.^30^ Therefore, publishing data on the impact of PSI-based interventions on patient harm can further support engagement and uptake of these interventions.

### Implications for practice

Strong leadership is needed at local and national levels to champion prescribing safety in primary care. The importance of taking a team approach to prescribing safety interventions in general practice must also be recognised. While the gradual change of employing pharmacists in general practice is encouraging, the study underscores a team-based approach to prescribing safety. Correspondingly, consistent training for pharmacists at undergraduate and postgraduate level to maximise their role in general practice needs to be considered, encompassing the use of PSI-based interventions, quality improvement, and root-cause analysis for identifying and preventing potentially hazardous prescribing. This training may also incorporate the value of sharing learning across practices, forming a learning network among practices, and complementing the public availability of prescribing safety data.

Publishing prescribing safety data according to localities and consistent with prescribing safety indicators would sustain PSI-based interventions and encourage friendly competitiveness in enhancing prescribing safety. Patients and the general public have an important role to play in prescribing safety, hence their involvement should be central to the publication of prescribing safety data alongside efforts to empower patients in understanding the role of pharmacists in general practice.

Importantly, policies and guidelines for prescribing safety should align throughout the healthcare system and facilitate communication between primary and secondary care. The endeavour to align policies could identify challenges and create communication channels for future joined-up care. PSI-based interventions should also align with national and local agendas and policies to be flexible to local strategies to demonstrate local relevance with national significance that will support sustainability.
